# Prevalence of vulvovaginal candidiasis in gynecological practices in Germany: A retrospective study of 954,186 patients

**DOI:** 10.18502/cmm.4.1.27

**Published:** 2018-03

**Authors:** Louis Jacob, Mara John, Matthias Kalder, Karel Kostev

**Affiliations:** 1 Faculty of Medicine, University of Paris 5, Paris, France; 2 Department of Gynecology and Obstetrics, Philipps University of Marburg, Marburg, Germany; 3 Department of Epidemiology, IQVIA, Frankfurt, Germany

**Keywords:** Germany, Gynecological practices, Prescription, Prevalence, Vulvovaginal candidiasis

## Abstract

**Background and Purpose:**

To the best of our knowledge, no information is available regarding the treatment of vulvovaginal candidiasis in gynecological practices. The goal of this study was to analyze the prevalence of vulvovaginal candidiasis (VVC) and the drugs prescribed for the treatment of this condition in women followed in gynecological practices in Germany.

**Materials and Methods:**

All the women followed in 262 gynecological practices between November 2014 and October 2016 were included in this study. The first outcome was the prevalence of patients diagnosed with VVC during this period. The second outcome was the prevalence of women with VVC who received an appropriate vaginal or systemic antimycotic prescription within 30 days after their first VVC diagnosis. Covariables included the use of gynecological/systemic antibiotics, consumption of oral/vaginal contraceptives, cancer, pregnancy, diabetes, and psychiatric diseases including depression, anxiety, and adjustment and somatoform disorders.

**Results:**

Between 2014 and 2016, 954,186 women were followed in gynecological practices, and 50,279 (5.3%) women were diagnosed with VVC during the same period. The use of gynecological antibiotics (OR=2.88), systemic antibiotics (OR=1.45), oral contraceptives (OR=1.74), and vaginal contraceptives (OR=1.84) were associated with an increase in the risk of VVC diagnosis. Cancer (OR=1.20) and pregnancy (OR=1.59) were additional risk factors. Approximately 75% of women diagnosed with VVC received an antimycotic prescription. The three most frequently prescribed drugs were clotrimazole (72%), fluconazole (14%), and nystatin (6%).

**Conclusion:**

More than 5% of women were diagnosed with VVC and the majority of them received an appropriate prescription.

## Introduction

Approximately 30-50% of women are affected by vulvovaginal candidiasis (VVC) at least once during their lifetime [1, 2]. Typical symptoms involve pruritus or burning sensation (27%) and dysuria (33%) [3, 4]. In most cases, VVC is caused by *Candida albicans*, *Candida glabrata,* or *Candida krusei*. The major risk factors for the development of VVC are lifestyle-related (e.g., frequency of sexual intercourse, contraception, or vaginal douching) [2, 3, 5]. Recent research has shown that these fungal infections have a considerable impact on the quality of life of women, underscoring the need for optimized management and treatment of patients diagnosed with VVC [6]. 

Only a few authors have analyzed the prevalence of VVC in Germany in recent years. In 2013, Foxman et al. investigated the prevalence of recurrent VVC in five European countries and the U.S. [2]. In Germany, more than 40% of women reported at least four acute episodes of VVC in a 12-month period. In 2015, Ruhnke et al. estimated that almost 2.5 million people are affected by recurrent vaginal candidiasis and that recurrent VVC is the second most frequent fungal infection in Germany after fungal skin diseases [7]. According to recent recommendations of the German Society of Gynecology and Obstetrics, all antimycotic agents for the treatment of VVC available on the market are equally effective, and the treatment of acute cases is largely successful [8].

Although the findings of these studies are important, there is a paucity of data regarding the prevalence of VVC in patients followed by gynecologists. Furthermore, to the best of our knowledge, no information is available regarding the treatment of these infections in this particular setting. Therefore, we aimed to analyze the prevalence and prescribed drugs for the treatment of VVC in women followed in gynecological practices in Germany.

## Materials and Methods

***Database***

In this retrospective study, we used data from the nationwide Disease Analyzer database (IQVIA). The Disease Analyzer database contains demographic, clinical, and pharmaceutical data anonymously obtained from a nationwide sample of general and specialist practices [9]. IQVIA regularly assesses the quality of information, and it has been previously found that the database is representative of primary care practices in Germany. The sample of practices is drawn based on the distribution of practices by region, specialty, and physician age groups in Germany. IQVIA updates the statistical plan, which forms the basis for the panel with the above-mentioned criteria, on a yearly basis based on the universe of all practices in Germany [9]. Moreover, the panel practices transmit patient data to IQVIA (Frankfurt) on a monthly basis. Before transmission, the data is encrypted for data protection purposes. Thus, the database includes only de-identified data in compliance with the regulations of German data protection law. No ethics approval is needed for retrospective data analyses with anonymous data in Germany. 

Finally, this database has already been used in studies focusing on infectious diseases and their associated treatments [10–12].

***Ethics statement***

German law allows the use of anonymous electronic medical records for research purposes under certain conditions. According to this legislation, it is not necessary to obtain informed consent from patients or approval from a medical ethics committee for this type of observational study since it contains no directly identifiable data. Therefore, no waiver of ethical approval was obtained from an Institutional Review Board or ethics committee. The authors had no access to any identifying information relating to the patients at any time during data analysis.

***Study population and outcomes***

Women aged 18 years or over and followed in 262 gynecological practices between November 2014 and October 2016 were included in this study. The first outcome was the prevalence of patients diagnosed with VVC (International Classification of Diseases, 10th revision [ICD-10]: B37.3) during this period. The second outcome was the prevalence of women with VVC who received an appropriate vaginal (Anatomical Therapeutic Chemical [ATC]: G01B0) or systemic antimycotic prescription (J02A0) within 30 days after the first VVC diagnosis. Covariables included the use of gynecological antibiotics (ATC: G01C), systemic antibiotics (ATC: J01), oral contraceptives (ATC: G01A, oral form or patch), and vaginal contraceptives (ATC: G01A, vaginal ring), cancer (ICD-10: C), pregnancy (ICD-10: Z32.1, Z33-36), diabetes (ICD-10: E10-14), and psychiatric diseases, including depression, anxiety, adjustment, and somatoform disorders (ICD-10: F32, F33, F41, F43, F45).

***Statistical analysis***

The prevalence of VVC, defined as the proportion of women diagnosed with VVC (denominator: all women who visited the gynecological practices), was analyzed. The association between VVC diagnosis and the predefined variables was studied using a multivariate logistic regression model. Finally, the percentages of women diagnosed with VVC receiving therapy were also analyzed. *P*-value less than 0.05 was considered significant. All the analysis were carried out using SAS 9.4 (SAS Institute, Cary, NC, USA).

## Results

**Figure 1:**
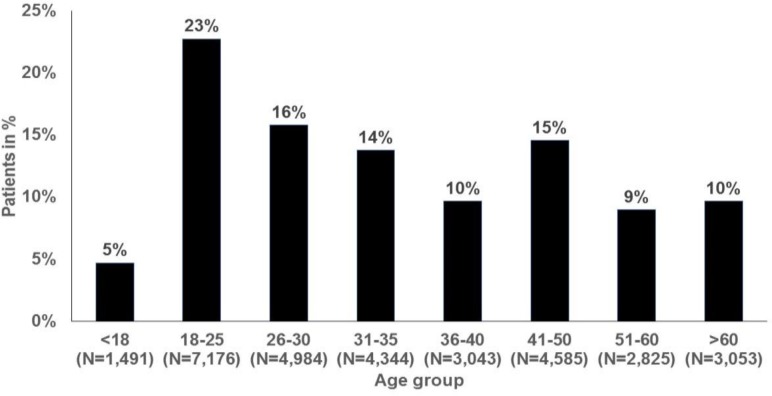
Age structure of women initially diagnosed with vulvovaginal candidiasis in 262 gynecology practices in Germany between November 2014 and October 2016 (N=31,501)

In total, 954,186 women were followed in 262 gynecological practices between 2014 and 2016 (mean age = 37.8±15.5 years), and 50,279 (5.3%) of these women were diagnosed with VVC during this period (Table 1). The highest prevalence rates were found in the age groups of 18-25 years (7.1%), 26-30 years (6.8%), and 31-35 years (6.9%). The age structure of women initially diagnosed with VVC is shown in Figure 1. Overall, 22.8%, 15.8%, and 13.8% of VVC patients were aged 18-25, 26-30, and 31-35 years old, respectively.

**Table 1:** Prevalence of vulvovaginal candidiasis in women followed in 262 gynecology practices in Germany between November 2014 and October 2016

**Age**	**Women with at least one visit to one of 262 gynecological practices**	**Women with a diagnosis of vulvovaginal candidiasis (N)** *	**Women with a diagnosis of vulvovaginal candidiasis (%)**
<18	40,142	1,712	4.3%
18-25	142,197	10,063	7.1%
26-30	112,542	7,631	6.8%
31-35	107,208	7,384	6.9%
36-40	88,680	5,543	6.3%
41-50	165,429	8,290	5.0%
51-60	137,408	4,779	3.5%
>60	160,580	4,877	3.0%
Total	954,186	50,279	5.3%

* Confirmed or ‘status post’ diagnoses are included.

**Table 2:** Association between vulvovaginal candidiasis and predefined variables in 31,501 women with vulvovaginal candidiasis and 31,501 age- and index date-matched controls in 262 gynecology practices

**Diagnoses or therapies within 12 months prior to index date**	**Candidiasis patients (%)**	**Non-candidiasis patients (%)**	**Odds Ratio (95% CI)**	**P-value**
Gynecological antibiotics	0.9	0.3	2.88 (2.26-3.67)	<0.001
Systemic antibiotics*	4.3	2.6	1.45 (1.32-1.58)	<0.001
Oral contraceptives (oral form or patch)	23.4	16.5	1.74 (1.66-1.81)	<0.001
Vaginal contraceptives (vaginal ring)	1.6	1.0	1.84 (1.59-2.12)	<0.001
Cancer (possible indicator for chemotherapy)**	2.0	1.6	1.20 (1.07-1.36)	0.003
Pregnancy	14.0	10.0	1.59 (1.52-1.68)	<0.001
Psychiatric diseases including depression, anxiety, and adjustment and somatoform disorders)***	4.9	3.9	1.08 (1.00-1.17)	0.067
Diabetes mellitus***	0.4	0.4	0.89 (0.69-1.15)	0.367

* Antibiotics prescribed in gynecological practices

** The majority of cancers were gynecological cancers

*** It is likely that the occurrence of non-gynecological diseases like diabetes and psychiatric diseases was underestimated

**Figure 2:**
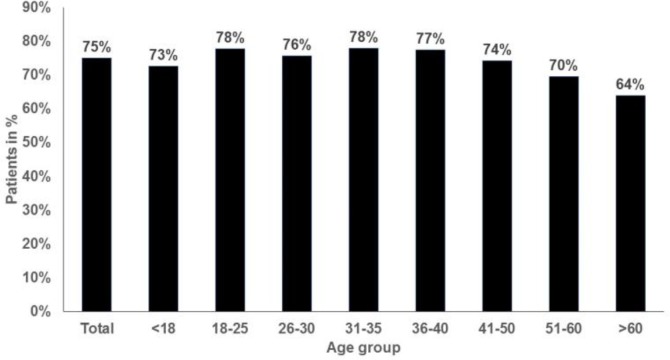
Proportion of women with vulvovaginal candidiasis receiving antimycotic drugs in 262 gynecology practices (N=31,501)

Note: due to large patient numbers, between-group differences are significant (*P<0.05*)

The results of the multivariate logistic regression model are displayed in Table 2. The use of gynecological antibiotics (OR=2.88), systemic antibiotics (OR=1.45), oral contraceptives (OR=1.74), and vaginal contraceptives (OR=1.84) were associated with an increase in the risk of VVC diagnosis. Cancer (OR=1.20) and pregnancy (OR=1.59) were additional risk factors. Finally, psychiatric diseases (i.e., depression, anxiety, and adjustment and somatoform disorders) and diabetes mellitus were not significantly associated with VVC.

**Figure 3:**
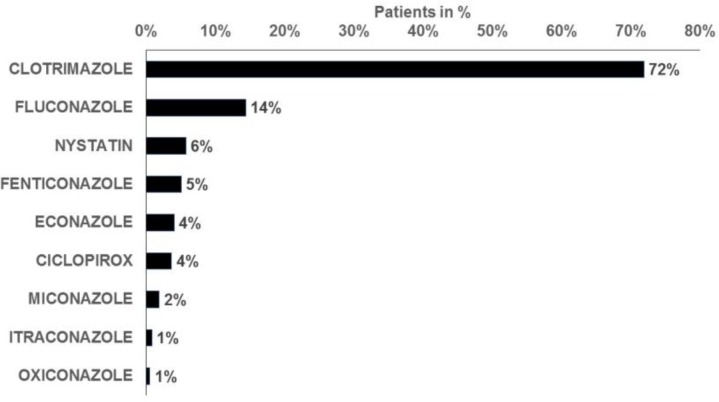
Antimycotic drugs prescribed for the treatment of vulvovaginal candidiasis in 262 gynecology practices (N=23,625)

Note: more than one drug can be prescribed for each patient

**Figure 4:**
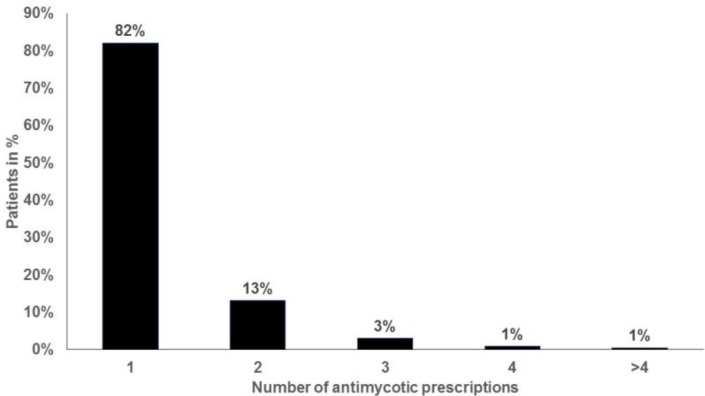
Number of antimycotic drug prescriptions per patient in 262 gynecology practices (N=23,625)

Approximately 75% of women diagnosed with VVC were prescribed an antimycotic agent, and the proportion of prescriptions issued ranged from 64% in women aged over 60 years to 78% in those aged 18-25 and 31-35 years (Figure 2). The three most frequently prescribed drugs were clotrimazole (72%), fluconazole (14%), and nystatin (6%; Figure 3). More than 95% of the women treated received the prescription on the first day of VVC diagnosis. Finally, the majority of VVC patients who required treatment received only one prescription (82%; Figure 4). 

## Discussion

The present retrospective study, including over 950,000 women followed in gynecological practices in Germany, showed that approximately 5% of the study population was diagnosed with VVC. VVC diagnosis was positively associated with antibiotic prescriptions, prescription of contraceptives, cancer diagnoses, and pregnancy. Finally, around 75% of VVC patients received an appropriate prescription. 

To the best of our knowledge, this is the first study to investigate the prevalence of VVC in gynecological practices in this country. Nonetheless, several authors have previously focused on the diagnosis of VVC in Germany. A study published in 2013, which was based on survey data from women in five European countries and the United States, estimated that VVC was reported at least once by 29-49% of the population [2]. The prevalence of VVC was about 45% in Germany. 

Each individual included in this study was given a questionnaire battery on yeast infections including questions on the presence of a medical diagnosis of VVC, the presence of recurrent VVC, the patient’s age at the first diagnosis of recurrent VVC, the number of years during which recurrent VVC was present, and the mean number of VVC episodes per year. The risk of multiple VVC recurrence was estimated at 10% in women with VVC aged 25 years and 25% in those aged 50 years, underlining the fact that the occurrence of multiple vaginal candidiasis infections is very common. Later, Ruhnke et al. focused on the estimated burden of fungal infections in Germany [7]. The authors found that 9.6 million (12%) people suffered from fungal infections in 2012, with fungal infections constituting more than 15 primary or recurrent fungal infections of different anatomical areas [7]. Approximately 6.7 and 2.5 million individuals were diagnosed with fungal skin diseases and recurrent VVC, respectively. 

Compared with the findings of Foxman et al. and Ruhnke et al. [2,7], VVC prevalence was much lower in our study. However, there are major differences that could explain this discrepancy. First, the work of Foxman et al. was based on an online survey consisting of questions on a variety of topics, which was given to more than 6,000 women aged 16-65 years [2]. The study of Ruhnke et al. was a meta-analysis of epidemiology studies published between 1975 and 2013, which reported fungal infection rates in Germany [7]. By contrast, we used data obtained from gynecological practices. Second, the definition of VVC varied between the three studies, as the authors of the first study looked at a past history of VVC [2], the authors of the second study investigated the diagnosis of recurrent VVC (at least four episodes in one year) [7], and we focused on any VVC episode diagnosed between 2014 and 2016. Furthermore, lifetime VVC likely includes symptomatic and asymptomatic cases, whereas VVC diagnosed in gynecological practices only includes symptomatic cases. This last difference might explain why the prevalence of VVC was around 5% in our work. Finally, since women with VVC can be also followed in general, dermatological, and internal practices, we might have underestimated the prevalence of VVC in Germany. Nonetheless, all studies underline the fact that women should be regularly examined by their gynecologist to diagnose and treat potential VVC infections.

A secondary outcome was that the use of gynecological/systemic antibiotics, the use of oral/vaginal contraceptives, cancer, and pregnancy were confirmed as risk factors for the development of VVC, as reported in the literature [13–21]. 

A case-control study including 1,585 patients estimated (after adjustment for age, marital status, and contraceptive method) that women reporting recent antibiotic use exhibited a 1.75-fold increase in their odds of being subsequently diagnosed with VVC [13]. More recently, in 2008, Xu et al. showed in 80 nonpregnant women aged 18-64 years that the prevalence of asymptomatic vaginal *Candida* colonization and the incidence of symptomatic VVC were significantly increased by the use of short courses of oral antibiotics [14]. As regards contraception, Ocak et al. found in 102 women that the prevalence of *Candida* species was around 15% in women receiving oral contraceptives, 12% in women who had had an intrauterine contraceptive device inserted, and 6% in women who did not use any contraceptive methods [15]. The most likely hypothesis is that both antibiotics and contraceptives can alter the vaginal microbiota, indirectly favoring the development of fungal infections. 

The impact of cancer on the risk of being diagnosed with fungal infections has been known for decades [16]. The emergence of these infectious species results from the immune system dysregulations caused by the tumor itself and by chemotherapy and other treatments [17–19]. Furthermore, Guzel et al. estimated in 2011 in 372 pregnant women that the prevalence of confirmed VVC was 37.4% and that of vaginal colonization was 11.3% in the study population [20], highlighting the fact that VVC is more commonly found during pregnancy. This high prevalence was corroborated in a more recent study of 80 healthy pregnant women from Bulgaria, as the authors diagnosed VVC in nearly 29% of them [21]. Interestingly, more than one out of five newborns born to these women also screened positive for *Candida* colonization. 

The present study concluded that an appropriate prescription had been received by approximately 75% of women diagnosed with VVC and followed by gynecologists in Germany. The three most frequently prescribed drugs were clotrimazole, fluconazole, and nystatin. The recent guidelines of the German Society of Gynecology and Obstetrics indicate that the treatment of VVC with the antimycotic agents available on the market is successful in most cases, and that polyenes such as nystatin or amphotericin B, imidazoles such as clotrimazole or miconazole, and oral triazoles such as fluconazole or itraconazole, are equally effective [8]. Cotrimoxazole (trimethoprim-sulfamethoxazole) was the most frequently prescribed drug, a finding that might be explained by the fact that it is a safe, effective, low-cost combination antibiotic prescribed for the treatment of a wide range of bacterial, parasitic, and fungal infections [22].

Although these findings are of particular importance, this study was subject to several limitations that should be mentioned at this point. First, since VVC diagnosis was based on ICD codes and not on biological data, the prevalence of VVC might have been underestimated. In addition, it was not possible to identify the causative pathogen or to determine its therapeutic susceptibility. Furthermore, there was no information on how VVC diagnoses were made, the symptoms exhibited by patients, and how treatment responses were evaluated by gynecologists. Second, even if women included in this study were also treated by general practitioners, we did not have access to the related data. Third, lifestyle-related factors were missing and we were thus unable to investigate their potential impact on the risk of being diagnosed with VVC. The main strength of this work was the number of patients and gynecologists included.

## Conclusion

More than 5% of women followed by gynecologists in Germany were diagnosed with VVC. Approximately three out of four women received antimycotic drugs (i.e., clotrimazole, fluconazole, or nystatin) for the treatment of VVC. Further research is needed to gain a better understanding of the factors associated with the prescription of these drugs in women diagnosed with VVC. 
